# Lake Topography and Wind Waves Determining Seasonal-Spatial Dynamics of Total Suspended Matter in Turbid Lake Taihu, China: Assessment Using Long-Term High-Resolution MERIS Data

**DOI:** 10.1371/journal.pone.0098055

**Published:** 2014-05-20

**Authors:** Yunlin Zhang, Kun Shi, Xiaohan Liu, Yongqiang Zhou, Boqiang Qin

**Affiliations:** 1 Taihu Lake Laboratory Ecosystem Station, State Key Laboratory of Lake Science and Environment, Nanjing Institute of Geography and Limnology, Chinese Academy of Sciences, Nanjing, China; 2 University of Chinese Academy of Sciences, Beijing, China; CNRS, France

## Abstract

Multiple comprehensive *in situ* bio-optical investigations were conducted from 2005 to 2010 and covered a large variability of total suspended matter (TSM) in Lake Taihu to calibrate and validate a TSM concentration estimation model based on Medium Resolution Imaging Spectrometer (MERIS) data. The estimation model of the TSM concentration in Lake Taihu was developed using top-of-atmosphere (TOA) radiance of MERIS image data at band 9 in combination with a regional empirical atmospheric correction model, which was strongly correlated with the *in situ* TSM concentration (*r* ^2^ = 0.720, *p*<0.001, and *n* = 73). The relative root mean square error (*RRMSE*) and mean relative error (*MRE*) were 36.9% and 31.6%, respectively, based on an independent validation dataset that produced reliable estimations of the TSM concentration. The developed algorithm was applied to 50 MERIS images from 2003 to 2011 to obtain a high spatial and temporal heterogeneity of TSM concentrations in Lake Taihu. Seasonally, the highest and lowest TSM concentrations were found in spring and autumn, respectively. Spatially, TSM concentrations were high in the southern part and center of the lake and low in Xukou Bay, East Lake Taihu. The lake topography, including the water depth and distance from the shore, had a significant effect on the TSM spatial distribution. A significant correlation was found between the daily average wind speed and TSM concentration (*r* ^2^ = 0.685, *p*<0.001, and *n* = 50), suggesting a critical role of wind speed in the TSM variations in Lake Taihu. In addition, a low TSM concentration was linked to the appearance of submerged aquatic vegetation (SAV). Therefore, TSM dynamics were controlled by the lake topography, wind-driven sediment resuspension and SAV distribution.

## Introduction

Large eutrophic shallow lakes are spatially and temporally complex environments because of the dynamic interactions of physical, chemical and biological factors [1]–[3]. Such lakes are often characterized by high and varying concentrations of total suspended matter (TSM) that result from terrestrial inputs and sediment resuspension [3], [4]. TSM dynamics plays a major role in many aspects of shallow lake ecology, including the underwater light climate [3], submerged aquatic vegetation (SAV) distribution [5], biomass primary production [6], and transport of nutrients, micropollutants, and heavy metals [4], [7], [8].

Thus, acquiring reliable TSM concentration temporal-spatial patterns is important for advancing our understanding of the ecosystem dynamics of large shallow lakes, developing effective and quantitative monitoring schemes, and improving water quality management. Ideally, measurements of TSM would be required to have high spatial and temporal resolution (order of tens of meters and tens of minutes, respectively). In recent years, a new option has been developed to acquire measurements of turbidity (a proxy of TSM) at a high temporal resolution (each minute or hour), and it uses high-frequency *in situ* sensors [9], [10]. However, observation networks are typically sparse (spacings of several kilometers), which is not compatible with the intrinsic small scale of TSM variability, which is induced by morphological features typically ranging from a few meters to kilometers. In addition, obtaining representative and successive measurements of environmental factors through traditional point sampling has proven to be problematic within these varying environments, especially when the data are extrapolated over large lake scales based on measurements at only a few sites.

Over the past three decades, TSM concentrations for oceans, coasts and lakes have been mapped by satellite water color data [11]–[14] and sensors such as the Sea-viewing Wide Field-of-view Sensor (SeaWiFS), Moderate Resolution Imaging Spectroradiometer (MODIS), Medium Resolution Imaging Spectrometer (MERIS), and Landsat ETM, all of which offer high spatial and temporal resolution.

Briefly, three main types of models are used to estimate TSM concentrations: (1) models that analyze the empirical or semi-empirical relationships between TSM concentration and a single wavelength or different wavelength ratios of the normalized water-leaving radiance (or remote sensing reflectance), such as the blue-green and blue-red wavelengths [11]–[15]; (2) models that employ semi-analytical approaches based on the relationship between the inherent optical properties (IOP) and water constituents [16], [17]; these models are used because TSM usually has strong absorption and backscattering properties in lake and coastal waters, and the TSM concentration can be derived from the backscattering coefficients and absorption coefficient of particles; and (3) comprehensive models that combine the empirical and semi-analytical approaches and cover a wide TSM range from <1 to >1,000 mg L^−1^ [18].

For lakes, especially extremely turbid lakes [19], there are fewer reports of remote sensing estimations of TSM than there are for oceans and coastal waters. In particular, for the large, shallow, and eutrophic Lake Taihu, there are few reports of TSM remote sensing, which is in contrast to the numerous studies investigating chlorophyll a remote sensing [20]–[23]. The long- and short-term dynamics of TSM concentration in extremely turbid, shallow lakes, such as Lake Taihu, have not been completely explored. There are frequent challenges to *in situ* sampling because of factors such as frequent sediment resuspension and strong winds [4]. Thus, remote sensing data would provide a useful representation of the TSM concentration over the entire lake because *in situ* water sample collection is not conducted during strong winds. Understanding the temporal-spatial variation in TSM concentrations in Lake Taihu is vital for the estimation of internal nutrient release [4], heavy metal adsorption and desorption [5], [8], and restoration of the SAV [24].

To address this concern, the objectives of our present study, which used Lake Taihu as an example of an extremely turbid and shallow inland lake, were to: (1) develop a regional MERIS atmospheric correction model based on synchronous remote sensing reflectance measurements, (2) develop a simple band model to estimate TSM concentrations based on MERIS image data, and (3) generate high spatial resolution maps of MERIS-derived TSM concentrations from 2003 to 2011 and assess the TSM seasonal-spatial and short-term dynamics.

## Materials and Methods

### Ethics statement

No specific permits were required for the described field studies. The location studied is not privately-owned or protected in any way, and the field studies did not involve endangered or protected species.

### Study sites and sampling schedule

**Figure 1:**
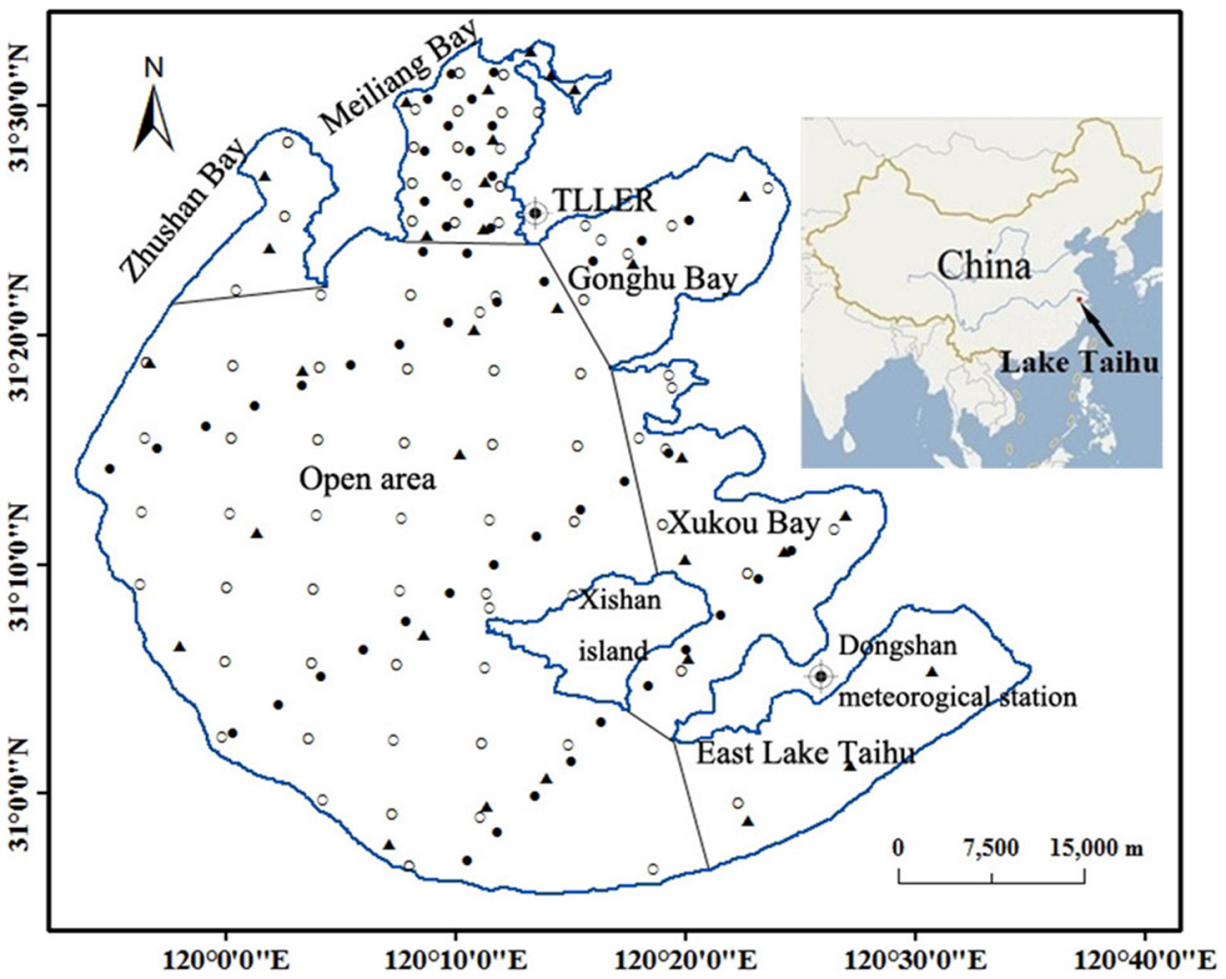
Distribution of sampling sites in Lake Taihu from 2005 to 2010 showing the three sources: (1) from 2005–2010, the sampling sites were scattered over the lake and used for long-term specific observations by the Taihu Lake Laboratory Ecosystem Research (TLLER); (2) from 2006 and 2007, the sampling sites were along 4 transects in four seasons; and (3) from 2007 and 2008 (November), the sampling sites were distributed around the lake. The three sources are denoted ▴, •, and ○, respectively, and the lake is divided into 6 sections.

The dataset in this study was composed of 425 water samples collected between 17 January 2005 and 14 January 2010 from three sources: (1) from 2005–2010, the sampling sites were scattered over the lake and used for long-term specific observations by the Taihu Lake Laboratory Ecosystem Research (TLLER); (2) from 2006 and 2007, the sampling sites were along 4 transects in four seasons; and (3) from 2007 and 2008 (November), the sampling sites were distributed around the lake; the three sources are denoted as ▴, •, and ○, respectively, in Fig. 1. The sampling frequency, time, number of samples, and detailed description are presented in Table S1. To accommodate the substantial spatial variation in the aquatic environments of Lake Taihu, we divided the lake into six sections: Meiliang Bay, Zhushan Bay, Gonghu Bay, open area, Xukou Bay and adjacent eastern coastal area, and East Lake Taihu (Fig. 1).

### TSM measurement

The TSM was filtered from water samples (100–500 ml according to the amount of particles) using Whatman GF/F fiberglass filters that had been pre-combusted at 550°C for 41 h; the filters were then dried at 105°C for 4 h and weighed using an electrobalance, which had an accuracy of 10^−2^ mg.

### Measurement of remote sensing reflectance

Measurements of downwelling radiance and upwelling total radiance were collected with an ASD field spectrometer (Analytical Devices, Inc., Boulder, CO) with a spectral response of 350 to 1,050 nm, spectral resolution of 3 nm, and sampling interval of 1 nm. The “above water method” was used to measure the water surface spectra [25].

The detailed measurement procedure was as follows. An optical fiber was positioned at nadir on a mount extending approximately 1 m away from the boat to reduce the influence of reflectance from the vessel on the collected spectra. The radiance spectra from the reference panel (*L*_p_(λ, 0^+^)), water (*L*_sw_(λ, 0^+^)), and sky (*L*_sky_(λ)) were measured at approximately 0.3 m above the water surface under clear sky conditions. At each sampling site, the spectra were measured 10 times to optimize the signal-to-noise ratio and reduce the error of the *in situ* measurements. Each spectrum was sampled at 90° azimuth from the sun and a nadir viewing angle of 40° to avoid the interference of the ship with the water surface and influence of direct sunlight. The water-leaving radiance *L*_w_(λ, 0^+^) can be derived from the following equation:
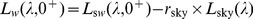
(1)where *L*_sw_(λ, 0^+^) is the upwelling radiance from water and *L*_sky_(λ) is the sky radiance measured at the same azimuth angle and 40° zenith angle. The *r*_sky_ is the spectral reflectance of skylight at the air–water interface, which is dependent upon wind speed. The values of *r*_sky_ ranged from 0.022 in calm weather to 0.025 at wind speeds of up to 5 m s^−1^ [25]. A constant value of 0.0245 was used in this study.

The incident downwelling irradiance *E*_d_(λ, 0^+^) was determined by measuring the radiance of the Lambertian reference panel *L*_p_(λ, 0^+^) as follows:
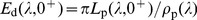
(2)where ρ_p_(λ) is the reflectance of the reference panel, which was accurately calibrated to 30%.

The remote sensing reflectance above the water surface *R*_rs_(λ, 0^+^) was calculated as the ratio of the water-leaving upwelling radiance *L*_w_(λ, 0^+^) to the incident downwelling irradiance *E*_d_(λ, 0^+^). We obtained a set of 347 *R*_rs_(λ, 0^+^) spectra.

### MERIS images

MERIS is a 68.5° field-of-view, push-broom, imaging spectrometer that measures the solar radiation reflected by the earth at a ground spatial resolution of 260×290 m. The central wavelengths of 15 spectral bands (B1–B15) are as follows: 412.5, 442.5, 490, 510, 560, 620, 665, 681, 709, 754, 761, 779, 865, 885, and 900 nm, with typical bandwidths of 10 nm [26].

Fifty high-quality MERIS images of Lake Taihu taken from 2003 to 2011 (Table S2) were downloaded from the European Space Agency (ESA) Earthnet Online site (http://earth.esa.int/). The images are level 1 processed, meaning that they have undergone calibration of the top-of-atmosphere (TOA) radiance. The images were processed using BEAM 4.8 software for geometric calibration and smile correction. The 50 high-quality MERIS images were used to estimate the TSM concentration and discuss TSM temporal-spatial dynamics in our study. The MERIS images were divided seasonally as follows: winter, December–February; spring, March–May; summer, June–August; and autumn, September–November (Table S2).

### Comparison of satellite and *in situ* data

The satellite data and *in situ* ground data should be concurrent within a period determined by the natural variation of the process being measured. We set the criterion for matching satellite and *in situ* observations to < = 2 days, to maximize the number of potential matching pairs between the satellite and *in situ* observations considering that wind speeds for the 2 day matching time are less than 3.2 m s^−1^ and change little within a mean value of 2.12±0.79 m s^−1^. As a result of the small wind speed, wind-driven sediment resuspension has almost no effect on the TSM concentration of surface water [4]. We defined a “match-up” as a data pair comprising a MERIS image and *in situ* data collocated in space (same pixel). Our criterion produced 147 “match-ups” of MERIS and water sample data, and 23 “match-ups” of MERIS and *R*_rs_(λ, 0^+^).

Subsequently, we divided the 147 “match-ups” into two parts: part 1, which was composed of 73 samples from 4 cruises in 2005, was used to calibrate TSM estimation model from MERIS image data; and part 2, which was composed of 74 samples from 6 cruises from 2007 to 2010, was used for validation of the model. The 23 “match-ups” were used to develop a regional atmospheric correction model.

### Wind speed data acquisition

To determine the relationship between wind speed and TSM concentration, and to consider the effect of lake topography on TSM concentration, we downloaded the long-term wind speed and direction data for Lake Taihu from 1956 to 2013, from the China Meteorological Data Sharing Service System (http://cdc.cma.gov.cn/home.do).

The wind data were collected at the Dongshan meteorological station site (31^o^4′N, 120^o^26′E), which is located at the peak of Dongshan Mountain at an elevation of 175 m (Fig. 1). The station is surrounded by Lake Taihu on three sides (Fig. 1), so the data from this site were considered to reflect the wind characteristics of the lake. The daily average wind speeds were calculated by reordering the wind speed data every 5 minutes and then using all of the available 5 minutes observations to calculate the wind speed for a day. The wind speed data had an accuracy of 0.1 m s^−1^. To analyze the effect of wind direction on the TSM concentration, the wind data were divided into four groups according to the wind direction: N (0°−45°, 315°–360°), E (45°–135°), S (135°–225°), W (225°–315°).

### Statistical analysis and assessment of accuracy

Statistical analyses including calculations of the mean, maximum, and minimum value and linear and non-linear regressions were performed using SPSS 17.0 software (Statistical Program for Social Sciences). To investigate the relationships between variables, we performed correlation analyses using SPSS software. Significance levels were reported to be significant (*p*<0.05) or not significant (*p*>0.05). The differences in parameters were assessed with independent sample *t*-tests (*p*<0.05).

To assess the accuracy of the algorithms, we used the fitting determination coefficient (*r* ^2^), relative root mean square error (*RRMSE*), and mean relative error (*MRE*). The parameters of *RRMSE* and *MRE* can be derived with the following equations:
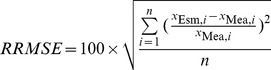
(3)

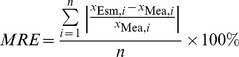
(4)where *x*_Esm, i_ and *x*_Mea, i_ are the estimated and measured values, respectively and *n* is the number of data points.

## Results

### Relationship between TSM and *R*_rs_(λ, 0^+^)

The relationship between TSM and *R*_rs_(λ, 0^+^) was determined using the datasets from the 7 cruises over the entire lake for which water samples and remote sensing reflectance measurements were collected (marked “w, rs” in column 4 of Table S1), which totaled 347 samples. There was a large variability in TSM concentrations, ranging from 8.20 to 285.60 mg L^−1^ with a mean of 61.90±53.09 mg L^−1^.

**Figure 2:**
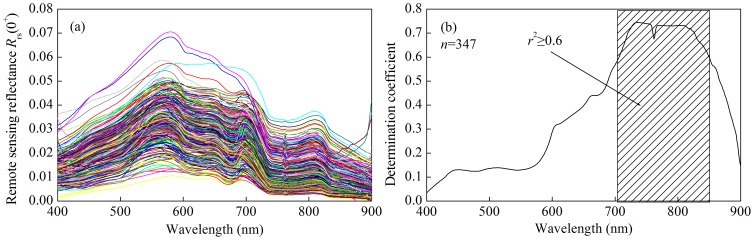
Remote sensing reflectance measurement of 347 sites from 7 cruises from 2006 to 2008 (a), and determination coefficient of the linear relationship between remote sensing reflectance and TSM concentration (b). The shaded area corresponds to determination coefficients higher than 0.6.

To find the optimal spectral bands for the TSM estimation, we performed a correlation analysis between the natural logarithm values of TSM and *R*_rs_(λ, 0^+^) (Fig. 2). There was a high determination coefficient >0.6 through the near-infrared wavelength range of 705 to 850 nm, which showed that the TSM concentration could be estimated accurately using *R*_rs_(λ, 0^+^) in this wavelength range; this result had previously been found for Lake Taihu and other turbid waters [14], [19], [27]. The high determination coefficient between the TSM concentration and *R*_rs_(λ, 0^+^) in the near-infrared region in Lake Taihu can be explained by the optical properties of the water, which are mainly controlled by the absorption of pure water and backscattering of TSM in the near-infrared region. The absorption coefficient spectrum of pure water is known, thus in the near-infrared region *R*_rs_(λ, 0^+^) is directly affected by the TSM.

The wavelength range from 705 to 850 nm corresponds to the 4 MERIS spectral bands B9–B12. Therefore, we used MERIS bands B9–B12 for the development of the TSM estimation model for Lake Taihu.

### Atmospheric correction

To assess the usefulness of the atmospheric correction of the BEAM 4.8 toolbox for MERIS, we conducted a correlation analysis between the *in situ* TSM concentration and two kinds of MERIS image data: (i) uncorrected top-of-atmosphere radiance (TOA) and (ii) atmospheric reflectance corrected by the trained artificial neural network (ANN) provided by the BEAM 4.8 software. There are three plug-in algorithms (Case-2 Regional Processer, Boreal Lakes Processor, and Eutrophic Lakes Processor) developed for MERIS level 1 data and provided by the basic BEAM 4.8 toolbox. However, the Boreal Lakes Processor and Case-2 Regional Processer were not designed for use in highly euphotic lake waters [28], [29]. Therefore, atmospheric correction was only conducted by the ANNs provided by the Eutrophic Lakes Processor (ELP).

The determination coefficients between the TSM concentration and uncorrected MERIS TOA were significantly higher (0.300–0.786) than the coefficients between the TSM concentration and MERIS reflectance that was corrected by ANN provided by the ELP of BEAM 4.8 software (0.001–0.148) (*t*-test, *p*<0.001) (Fig. S1), which showed that the atmospheric correction of the BEAM 4.8 toolbox for MERIS was invalid for the extremely turbid Lake Taihu; this result was confirmed in other turbid and highly euphotic lake waters [26], [30], [31].

**Figure 3:**
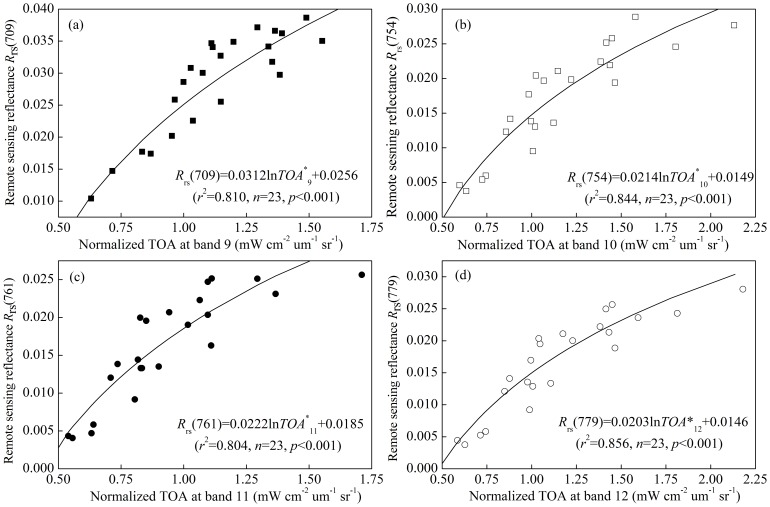
Correlations between the normalized top-of-atmosphere radiance (TOA) and remote sensing reflectance at the four MERIS bands 9 (a), 10 (b), 11 (c) and 12 (d).

Atmospheric correction methods over water often assume a zero water reflectance in the near infrared wavelength; however, for high TSM values (>50 mg L^−1^) this assumption becomes invalid [26], [32], [33]. The mean value TSM concentration among the 347 samples was 61.90±53.09 mg L^−1^, which exceeded the 50 mg L^−1^ threshold and indicated that the atmospheric correction of the ANN from ELP was invalid [33]. Therefore, for our Lake Taihu study area, this assumption should not be applied; instead, a regional atmospheric correction model should be developed based on the measured *R*_rs_(λ, 0^+^). An analysis of the correlations between the normalized TOA of the MERIS data and measured *R*_rs_(λ, 0^+^) was conducted for the 23 match-ups of the MERIS bands 9–12 (Fig. 3). There was a highly significant logarithmic algorithm between the TOA and measured *R*_rs_(λ, 0^+^) for all four bands. Therefore, we developed an empirical regional atmospheric correction to correct the TOA to *R*_rs_(λ, 0^+^) for further TSM estimation model calibrations and validations. Of course, future works are required to collect additional match-up data in similar waters to develop a more accurate regional atmospheric correction model.

### Estimation model of TSM concentration: calibration and validation

To calibrate and validate our TSM estimation model, we used the independent datasets from the different cruises. The calibration dataset was composed of 73 samples collected during four cruises in 2005: 17 January, 20–22 February, 17–19 November, and 17 December. The validation dataset was composed of 74 samples collected during six cruises: 7–9 January, 8–21 November, 19–21 November in 2007; 10–21 November in 2008; 13 January in 2009; and 14 January in 2010 (Table S1).

In the calibration dataset, the TSM concentration ranged from 8.80 to 321.38 mg L^−1^, with a mean of 81.48±62.07 mg L^−1^, and there were significant temporal and spatial differences. To find the best wavelength band and fitting model by which to estimate the TSM concentration in the extremely turbid Lake Taihu, the simple band model using the four band channels of the MERIS data were tested for their correlation with the TSM concentration based on the linear and power algorithms. The determination coefficients of bands B9, B10, and B12 using the linear relationship and band B9 using the power algorithm were higher than 0.7 and significantly higher than the coefficients of other bands and algorithms (*t*-test, *p*<0.001) (Fig. S2a). In addition, the *MRE* and *RRMSE* of band B9 using the power algorithm were significant lower than the values from other bands and algorithms (*t*-test, *p*<0.001) (Fig. S2b, S2c). Therefore, by combining the determination coefficient and *MRE* and *RRMSE* values, the power algorithm based on band B9 was recommended for the estimation of the TSM concentration in Lake Taihu.

**Figure 4:**
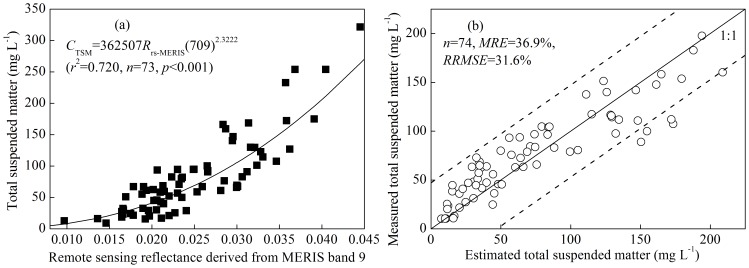
Calibration (a) and validation (b) of the simple single-band model to estimate the TSM concentration in turbid Lake Taihu based on the remote sensing reflectance derived from MERIS band 9 and a regional atmospheric correction model using the top-of-atmosphere radiance (TOA) data. The dashed lines indicate two *RMSEs* of the TSM estimation.

There was a highly significant power function between the remote sensing reflectance derived from the MERIS band 9 (*R*_rs-MERIS_(709)) and TSM concentration (*C*_TSM_) (Fig. 4a). Thus, the simple optical model of the TSM concentration estimation in the highly turbid Lake Taihu has the following form:
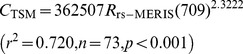
(5)


Validation is fundamental to the development of an estimation procedure to evaluate the overall reliability of the retrieval scheme and characterize the uncertainty associated with the estimated values. The model performance must be evaluated using a validation dataset that has not been used in the calibration phase. We evaluated the performance of the simple optical model using an independent validation dataset of 74 samples to further understand its applicability to the estimation of TSM concentration. To validate our model, we used values of TSM concentrations from 7.54 to 208.87 mg L^−1^, which had a mean of 74.53±55.13 mg L^−1^ that fell well into the range and mean value of the TSM concentrations used to calibrate the model.

Comparisons of the measured and estimated TSM concentrations using the calibrated simple optical model showed that these values were consistent and had a highly significant linear relationship (*r* ^2^ = 0.824). The measured and estimated TSM concentrations were distributed along the 1∶1 line (Fig. 4b), indicating that the simple optical model could be used for the extremely turbid waters of Lake Taihu. In addition, the *MRE* and *RRMSE* values of the validation data were close to or even lower than the values of the calibration dataset, which indicated that the developed TSM estimation model had a good universality and applicability. The simple band model was calibrated using data collected in 2005, and the specific form of this model expressed by the power function in Eq. (5) was applied to predict the TSM concentrations for an independent dataset collected from 2007 to 2010. This algorithm did not require further optimization from site-specific parameterizations to accurately estimate the TSM concentration in Lake Taihu, even with its widely varying TSM concentrations and bio-optical characteristics (Fig. 2).

**Figure 5:**
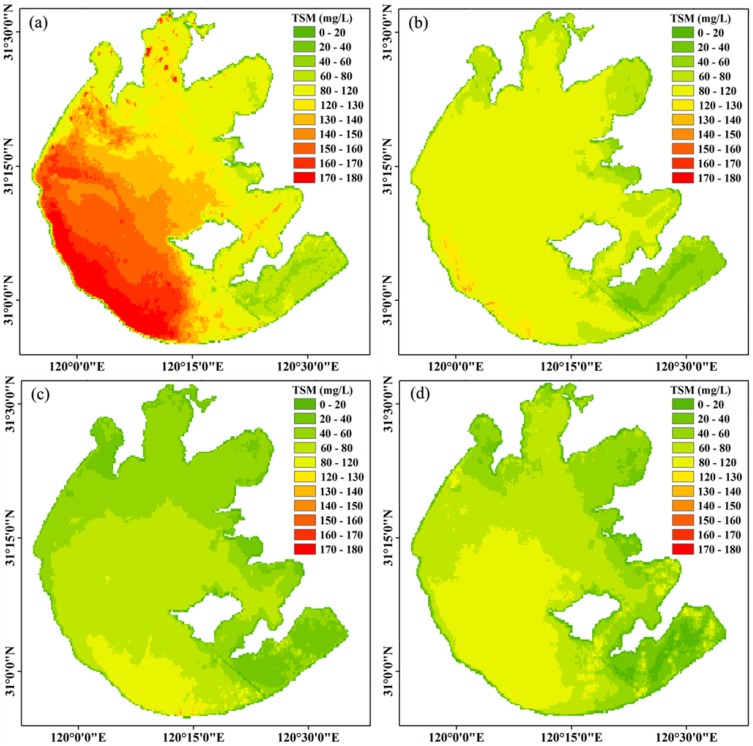
Seasonal variations in the MERIS-derived TSM concentration in Lake Taihu: spring (*n* = 10) (a), summer (*n* = 12) (b), autumn (n = 11) (c), and winter (*n* = 17) (d).

The MERIS-derived TSM concentrations of the 50 images from 2003 to 2011 were used to obtain the seasonal variation according to the seasonal distribution of MERIS images in Table S2. The TSM concentrations were significantly higher in spring and summer than in autumn and winter (*t*-test, *p*<0.01) (Table 1, Fig. 5), with a mean concentration in spring and summer (112.78±84.03 mg L^−1^) that was almost twice the mean in autumn and winter (61.87±54.66 mg L^−1^). There were no significant differences in the TSM concentration between spring and summer (*t*-test, *p* = 0.70) or between autumn and winter (*t*-test, *p* = 0.15).

**Table 1:** Seasonal and spatial variations in the MERIS-derived TSM concentrations for the entire lake, and each of the six regions of Lake Taihu from 2003–2011.

		Minimum	Maximum	Median	Mean	Standard deviation
Entire lake	All (*n* = 50)	11.46	314.82	54.98	84.27	72.97
	Spring (*n* = 10)	19.08	269.34	102.46	122.69	79.45
	Summer (*n* = 12)	14.08	314.82	75.04	104.53	90.28
	Autumn (*n* = 11)	15.96	183.44	49.26	56.45	46.62
	Winter (*n* = 17)	11.46	208.45	40.67	64.88	59.74
Meiliang Bay (*n* = 50)		3.54	233.62	57.26	72.07	68.34
Gonghu Bay (*n* = 50)		0.83	244.27	37.35	65.97	68.23
Zhushan Bay (*n* = 50)		1.22	234.63	39.77	62.34	66.14
Open area (*n* = 50)		15.80	346.21	66.27	95.71	79.38
Xukou Bay (*n* = 50)		0.91	288.30	46.75	70.04	68.71
East Lake Taihu (*n* = 50)		0.71	257.36	29.08	48.80	56.38

Data presented are minimum, maximum, median, mean and standard deviation (unit: mg L^−1^).

### Spatial distribution of the TSM concentration in Lake Taihu

**Figure 6:**
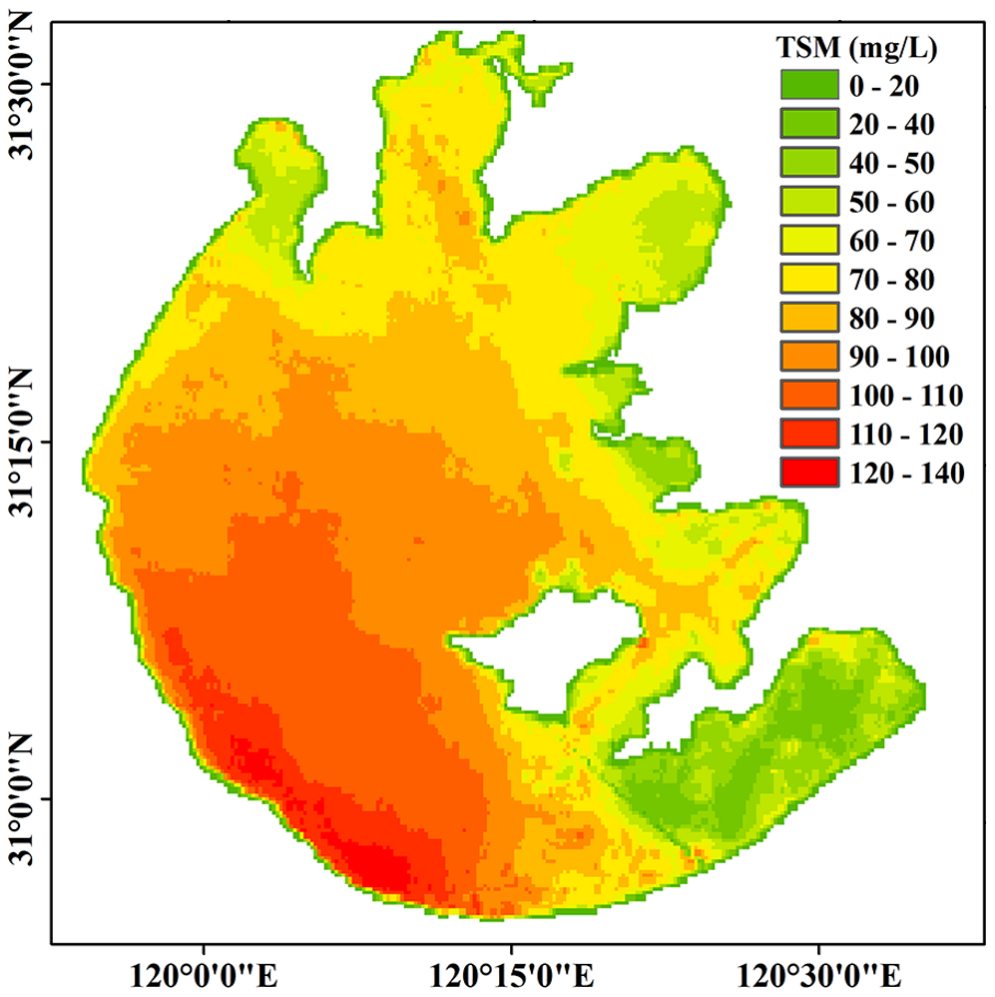
Spatial distribution of the MERIS-derived TSM concentration in Lake Taihu average from 50 images from 2003 to 2011.

There was large spatial variability of the MERIS-derived TSM concentration from the 50 images taken of the large shallow Lake Taihu (Fig. 6), and the statistical parameters of the TSM concentration for the entire lake and each of its six regions were calculated (Table 1). The TSM concentration of the entire lake ranged from 11.46 to 314.82 mg L^−1^, with a mean of 84.27±72.97 mg L^−1^. These values confirmed that Lake Taihu was generally highly turbid, which was consistent with the results of several previous studies [19], [24], [32].

The highest TSM concentration was in the southwest open area, followed by Meiliang Bay, Xukou Bay, Gonghu Bay and Zhushan Bay (Fig. 6, Table 1). In Meiliang Bay, Gonghu Bay and Zhushan Bay, the TSM concentration increased from the inner bay to the outer regions. The lowest TSM concentration was in East Lake Taihu (Fig. 6, Table 1), whereas the TSM concentrations were significantly higher in the open areas than in Meiliang Bay, Gonghu Bay, Zhushan Bay or Xukou Bay (*t*-test, *p*<0.05); the TSM concentration in each of these four bays was significantly higher than in East Lake Taihu (*t*-test, *p*<0.05) (Fig. 6, Table 1). There were no significant spatial differences in the TSM concentration between the four bays (Meiliang Bay, Gonghu Bay, Zhushan Bay and Xukou Bay) (Table 1) (*t*-test, *p*>0.05).

## Discussion

### Effect of lake topography on TSM concentration

There was a consistent spatial distribution of TSM concentration and water depth in which higher TSM concentrations corresponded to deeper water depths (Figs. 6, S3a). Furthermore, there was a significant positive correlation between the TSM concentration and water depth for every pixel of MERIS data (Fig. S3b). As a result of the high spatial resolution, the MERIS data allowed us to analyze the interrelationship between the water depth and TSM concentration.

There were several different mechanisms to explain the spatial consistency of TSM concentration and water depth. First, the shallow waters were in the littoral zones and lake bays, whereas the deep waters were in the open lake area. In the littoral zones and lake bays, the wind fetch was shorter than in the open area, which decreased the intensity of wind waves [34], [35] and wind-driven sediment resuspension [4] and resulted in a lower TSM concentration than in the open area. This situation in Lake Taihu is in contrast with the ocean, where the higher TSM concentrations are in the shallow coastal waters because of sediment resuspension and river input. In addition, there are lower TSM concentrations in the deep open ocean [13], [18]. Therefore, a significant negative correlation was found between water depth and TSM concentration in the coastal areas and open ocean [36], [37]. The difference between large shallow lakes and oceans was partly attributed to the entirety of Lake Taihu suffering sediment resuspension because of its shallow water depth and high dynamic ratio (the square root of the surface area divided by the mean depth), which was as high as 25.6 km m^−1^ (100% of the lake bottom subject to sediment resuspension) [38], whereas only part of the coastal waters suffered from sediment resuspension in the ocean. The second factor explaining the consistency of the positive correlation relationship between TSM and depth in Lake Taihu was that the SAV distributed in the shallow parts of the lake [39], inhibited sediment resuspension [3], [40] and resulted in a lower TSM concentration in the shallow regions compared to the deeper parts of the lake.

To further consider the effect of lake topography on the TSM concentration, we calculated the distance between each pixel and the shore in four directions (N, E, S, and W). There were significant positive linear relationships between the TSM concentration and distance to the north and east shores (*r* ^2^ = 0.57 for N and *r* ^2^ = 0.32 for E) but no significant correlations between the TSM concentration and distances to the south and west shores, which indicated that the TSM concentration was easily affected by N and E winds and not S and W winds. We further analyzed the frequency distribution of 16 wind directions of the daily maximal wind speed from January 1956 to August 2013 at Dongshan meteorogical station. The dominant wind directions in Lake Taihu were NNW and ESE (Fig. S4), which caused longer wind fetches for N and E directions than for S and W. Therefore, the TSM concentration in Lake Taihu was easily affected by the N and E winds.

**Figure 7:**
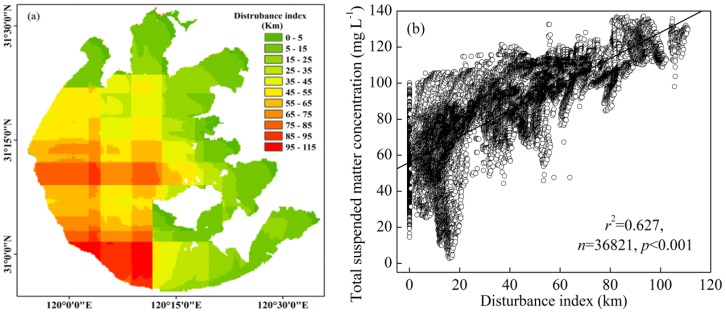
Spatial distribution of the disturbance index in Lake Taihu (a), and linear relationship between the disturbance index and total suspended matter concentration (b).

We here newly define a disturbance index, in which the degree of wind-driven disturbance of water is the sum of the distances between the pixels for the two directions that were most easily affected by wind (N and E in this study). The spatial distribution of the disturbance index corresponded well to the spatial distribution of the TSM concentration (Fig. 7a). Furthermore, there was a highly significant positive linear relationship between the disturbance index and TSM concentration (Fig. 7b), which indicated that the disturbance index could be used to reflect the degree of turbidity in Lake Taihu. Therefore, the simplicity to calculate the disturbance index has potential applications in numerous fields, including the study of spatial dynamics of TSM concentrations and particulate nutrients, investigations of the relationship between TSM concentration, particulate nutrients and wind waves, and assessments of the sedimentation and suspension flux.

### Effect of wind-driven sediment resuspension on TSM concentration

**Figure 8:**
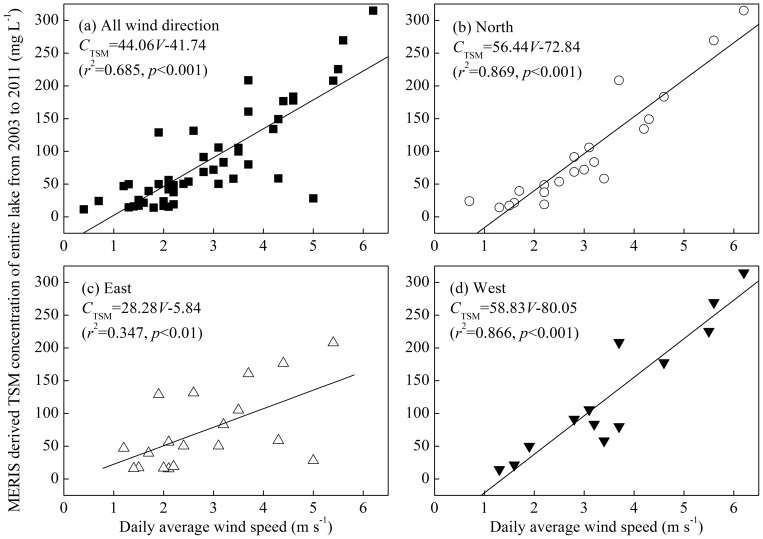
Linear relationships between daily average wind speed and MERIS-derived TSM concentration of the entire lake from 2003 to 2011 under all of the wind directions (a), north winds (b), east winds (c), and west winds (d). The linear relationship under southerly winds was not significant, so it is not presented.

For the entire lake, there were significant linear relationships between the daily average wind speed and MERIS-derived TSM concentration when all of the wind directions were considered together and when the N, E and W winds were considered individually (Fig. 8); there was no significant relationship for the S wind. This result showed that the wind-driven sediment resuspension significantly increased the TSM concentration of the water column, as has been widely observed in shallow lakes or estuaries based on *in situ* sample collections, model simulations, and remote sensing methods [4], [9], [41]–[43].

However, the increase of TSM concentration with wind speed was different for the different wind directions. For the S wind, there was no significant linear relationship between the daily average wind speed and TSM concentration, indicating that the TSM concentration was not driven by the S wind (Fig. 8). However, the TSM concentration was easily driven by the N, E and W winds (Fig. 8). These results showing the significant relationships with winds from the west slightly differed from the effects of lake topography on TSM concentrations, which showed that no significant positive correlation occurred between the TSM concentration and distances to the west, which represented the wind fetch driven by the W wind. These differences could be attributed to two aspects: 1) SAV distribution in the eastern lake regions [39], [44], [45] and 2) low frequency of the W wind in Lake Taihu (Fig. S4).

Numerous studies have revealed that wind-driven sediment resuspension was the primary force controlling the short-term variability in TSM concentration [41], [43]. The advective entrainment of surficial sediments into the water column increased the TSM concentration by wind events and generally occurred over time scales of hours to days.

**Figure 9:**
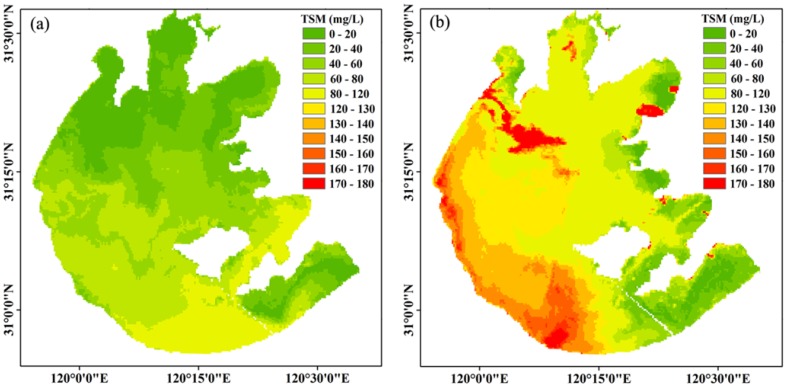
Spatial distribution of TSM concentration on two sequential days with short-term increasing wind speed over 24 hours. April 24, which had low TSM concentrations in the northern lake regions under a low wind speed (2.1 m s^−1^) (a), and April 25, which had a clear directional TSM concentration increase in the northern lake regions resulting from wind-driven sediment resuspension by southerly winds and a high wind speed (3.5 m s^−1^) (b).

Our data for Lake Taihu for the spatial distribution of TSM concentrations for two sequential days with short-term increasing wind speeds is shown in Fig. 9. Over 24 hours, the wind speed increased from 2.1 m s^−1^ on April 24 to 3.5 m s^−1^ on April 25, 2008, and the wind direction veered from S to N. There was a clear directional TSM signal followed by a higher concentration caused by sediment resuspension when the daily average wind increased. The image from April 25 (Fig. 9b) showed the highest TSM concentrations in the downwind northern part of Lake Taihu because the fetch was long enough to allow resuspension or horizontal advection. On April 25, the TSM concentration in Meiliang Bay, Gonghu Bay and Zhushan Bay in the downwind northern part of Lake Taihu increased by approximately 4 to 7 times that of April 24 and 2.35 times of that of the open area. However, the TSM only had a negligible increase in Xukou Bay and East Lake Taihu near the southeastern lake shore compared to April 24 (Fig. 9). High winds and the subsequent strong wave action caused the TSM concentration to increase because of the wind-driven sediment resuspension. Similar results were also observed in other shallow lakes or estuaries [9], [42], [43]. For example, the TSM concentration significantly decreased when the daily average wind speed decreased from 5.3 m s^−1^ on May 10 to 3.2 m s^−1^ on May 11, 2006 in Lake Markermeer, a shallow lake in the Netherlands with an area of 680 km^2^ and average depth of 3.6 m [43].

In addition, there was a potential application for our results in the TSM concentrations prediction in Lake Taihu and similar large shallow lakes. The empirical relationship (Fig. 8a) was developed based on 25,670 pixels of the entire Lake Taihu; therefore, the results were not affected by any particular position, such as the bay or open water. Therefore, the empirical relationship could be used to predict the TSM concentrations for high wind conditions (such as for a typhoon) using the wind speed without TSM concentration measurements because the water samples used to measure the TSM concentrations were collected in fair-weather conditions.

However, studies have indicated that the occurrence and magnitude of sediment resuspension in shallow lakes are also determined by factors other than wind speed, such as water depth, fetch, wind direction, SAV biomass, resuspension history and sediment structure/cohesiveness [3], [40], [43], [46], [47]. To further determine the response of the TSM concentrations of different regions of Lake Taihu to different wind directions, we calculated the determination coefficient and significance level of linear relationship between the daily average wind speed and TSM concentration for each of the six lake regions under different directions (Table 2). The TSM was easily driven by wind in Meiliang Bay, Gonghu Bay, Zhushan Bay and the open area, with these regions having the highest determination coefficients (Table 2). In contrast, TSM was not easily driven by E or S winds in Xukou Bay (Table 2), or in East Lake Taihu because there were no significant linear relationships between the daily average wind speed and TSM concentration (Table 2). Evidently the resuspension mechanisms for East Lake Taihu were different than those operating in Meiliang Bay, Gonghu Bay, Zhushan Bay, and the open area. East Lake Taihu was surrounded by the shore (Fig. 1), and was covered by dense SAV [39], [44], [45], which meant that the TSM concentrations were not easily affected by wind-driven sediment resuspension.

**Table 2:** Linear determination coefficients (and their significance level) between the daily average wind speed and TSM concentration in the six regions of Lake Taihu under different wind directions: north: 0°–45°, 315°–360°; east: 45°–135°; south: 135°–225°; and east: 225°–315°.

	All directions	North	East	South	West
Meiliang Bay	0.671*	0.872*	0.336**	0.254^ns^	0.862*
Gonghu Bay	0.650*	0.865*	0.324***	0.240^ns^	0.793*
Zhushan Bay	0.663*	0.875*	0.329***	0.225^ns^	0.829*
Open area	0.685*	0.858*	0.358**	0.257^ns^	0.875*
Xukou Bay	0.622*	0.798*	0.277***	0.176^ns^	0.848*
East Lake Taihu	0.485*	0.745*	0.121^ns^	0.116^ns^	0.685*

*: *p*<0.001;

**: *p*<0.01;

***: *p*<0.05;

ns : *p*>0.05 (not significant).

**Figure 10:**
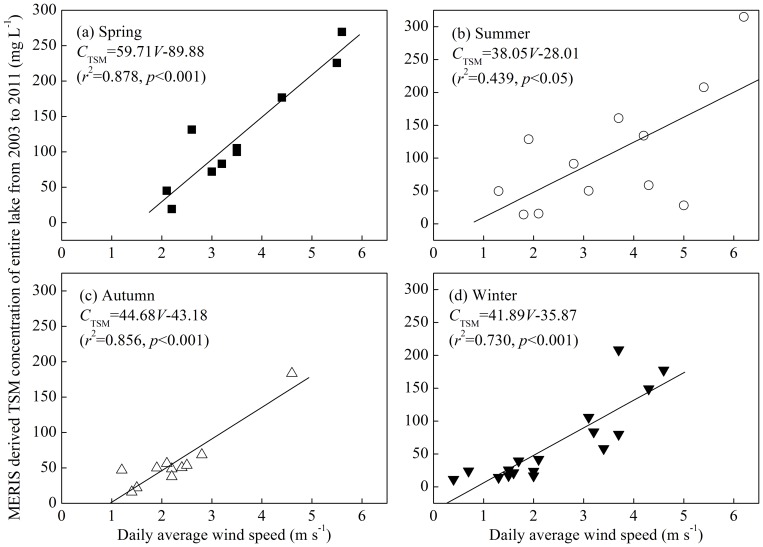
Linear relationships between the daily average wind speed and MERIS-derived TSM concentration of the entire lake from 2003 to 2011 for four seasons: spring (a), summer (b), autumn (c) and winter (d).

Wind-driven sediment resuspension not only controlled the spatial distribution of the TSM but also its seasonal variation. There were significant linear relationships between the daily average wind speed and TSM concentration for all four seasons (Fig. 10). However, the determination coefficient and significance level in summer were significantly lower than in the other three seasons, indicating there were factors other than wind-driven sediment resuspension that controlled the TSM concentration in summer. Over the past 15 years, algal blooms have frequently occurred in Lake Taihu in summer, especially in Meiliang Bay and Zhushan Bay in the north [48], [49]. The accumulation of floating algal blooms increased the TSM concentration; therefore, during summer blooms, the TSM concentration was not highly correlated with the daily average wind speed. However, in the other three seasons, the algal blooms rarely occurred and only had a slight effect on the TSM concentration. Therefore, the TSM concentration in spring, autumn and winter was predominantly determined by wind-driven sediment resuspension.

In Lake Taihu, the water column becomes well mixed and the TSM can be resuspended throughout the entire column because of the lake topography and high dynamic ratio (25.6 km m^−1^). Although only the surface TSM signal would be received by the satellite, the patterns of TSM concentrations at the bottom and water surface can be similar, with the exception of higher concentrations at the water-sediment interface near the bottom. Therefore, the surface MERIS-derived TSM concentrations could reflect the water column under well-mixed conditions. However, a three-dimensional model should still be a good alternative for extrapolating remotely sensed surface TSM concentrations for profiles with steep concentration gradients to the bottom. In addition, remote sensing data for windy (and often cloudy) conditions were not available.

### Interaction between the SAV distribution and TSM concentration

Numerous previous studies have shown a large spatial difference in the SAV distribution in Lake Taihu, with most of the SAV being widely distributed in the littoral region of Gonghu Bay, Xukou Bay and East Lake Taihu [35], [39], [44], [45]. This distribution corresponded to TSM concentrations of less than 30 mg L^−1^ and a water depth of less than 2.0 m (Fig. 6 and Fig. S3). Similar results were found in other studies of shallow lakes and coastal lagoons [1], [5], [46]. For example, in Lake Okeechobee, which is a shallow subtropical lake in USA, dense SAV was only found where the TSM was <20–30 mg L^−1^ and water depth was <2.0 m [5].

The shallow water depth in the littoral region of Gonghu Bay, Xukou Bay and East Lake Taihu corresponded to a lower TSM concentration than in the open area, which favored the growth of the SAV. The growth and distribution of the SAV can improve the underwater light climate by allelopathy and suppress the resuspension of the bottom sediment, which would decrease the TSM concentration [3], [40]. In addition, the SAV affect water quality by providing nutrient uptake, shade, and habitat for zooplankton and small fish [50]. In the bays of Lake Taihu, the SAV growth and distribution exerted a positive feedback in which resurging SAV reduced the sediment resuspension and created a clearer water column for SAV growth [46], [51]. In contrast, negative feedback occurred when sediment resuspension in the open area without SAV prevented recolonization [52] because of waves or currents preventing the establishment of propagules [53] or greater light requirements for recolonizing SAV [54]. Therefore, the SAV growth and lower TSM concentration had a reciprocal causation.

Light availability was the core interaction between the SAV distribution and TSM concentration. Several studies have demonstrated that in shallow lakes, light was the key factor that determined the maximum colonization depth of the SAV [52], [54], [55]. Because wind-driven sediment resuspension decisively determined the underwater light climate in Lake Taihu [3], [24], light availability and wave exposure are strongly interrelated. As a consequence, the influence of these two factors on the growth of SAV cannot be distinguished without considering the key features of the complex interplay between site-specific morphometry and dynamically changing wind fields. The situation is similar to other shallow lakes where light is primarily determined by sediment resuspension.

## Conclusions

To reduce the difficulty of atmospheric correction, our study first developed a regional empirical atmospheric correction model connecting the measured *R*_rs_(λ, 0^+^) and TOA radiance of the pixels of synchronous MERIS images. A simple band model of TSM estimation was initially calibrated using *in situ* TSM concentrations from 2005 and MERIS band 9 TOA radiance corrected by the former regional empirical atmospheric correction model, which had a relatively high modeling accuracy (*r* ^2^ = 0.720, *p*<0.001, and *n* = 73). The model was validated and further evaluated using an independent dataset (*n* = 74) from 2007–2010 and produced low predictive errors (*MRE* = 36.9%, *RRMSE* = 31.6%).

The effects of lake topography, wind-driven sediment resuspension and SAV distribution on TSM concentration were evaluated according to the temporal-spatial pattern of the MERIS-derived TSM concentrations. There were significant positive correlations between TSM concentration and water depth, the disturbance index of every pixel of the MERIS data, indicating that the TSM concentration was first determined by the lake topography in this shallow lake. There was a significant correlation between the average TSM concentration of the entire lake and daily average wind speeds (*r* ^2^ = 0.685, *p*<0.001, and *n* = 50), which suggested that wind speeds performed a critical role in the TSM variations in Lake Taihu. In addition, the low TSM concentration was linked to the SAV distribution, which showed the interaction of TSM and SAV growth, meaning that the SAV distribution in the shallower waters caused the lower TSM concentration, whereas the lower TSM concentration favored SAV growth. Therefore, the TSM dynamics were jointly controlled by lake topography, wind waves and SAV distribution in Lake Taihu.

## Supporting Information

Figure S1 Comparison of the determination coefficients between TSM concentration and the 15 bands of MERIS data for: (i) MERIS uncorrected top-of atmosphere radiance (TOA), and (ii) MERIS atmospheric reflectance corrected by the trained artificial neural network (ANN) provided by Eutrophic Lakes Processors (ELP) of Beam 4.8 software. There is no data at B11, B14 and B15 for ANN because the three bands are used for atmospheric correction.
(TIF)

Figure S2 Comparison of the determination coefficient (a), mean relative error (*MRE*) (b), and relative root mean square error (*RRMSE*) (c) using the linear and power algorithm for MERIS bands B 9–12.
(TIF)

Figure S3 Spatial distribution of water depth in Lake Taihu (a), and linear relationship between water depth and TSM (b).
(TIF)

Figure S4 Rose diagram frequency distribution of 16 wind directions of daily maximal wind speed from January 1956 to August 2013 at Dongshan meteorogical station.
(TIF)

Table S1 Cruise sampling date, number of samples, distribution of sampling sites, type of data, and MERIS data date for 14 cruises in Lake Taihu during 2005–2010.
(DOCX)

Table S2 Seasonal distribution of MERIS images in Lake Taihu during 2003–2011. The dates of the images are shown in 8 digits: year, month, and date.
(DOCX)
