# Supplementary figures and images for: Lake Topography and Wind Waves Determining Seasonal-Spatial Dynamics of Total Suspended Matter in Turbid Lake Taihu, China: Assessment Using Long-Term High-Resolution MERIS Data
Source: PLoS One. 2014 May 20;9(5):e98055. doi: 10.1371/journal.pone.0098055 (PMC4028274; doi:10.1371/journal.pone.0098055)

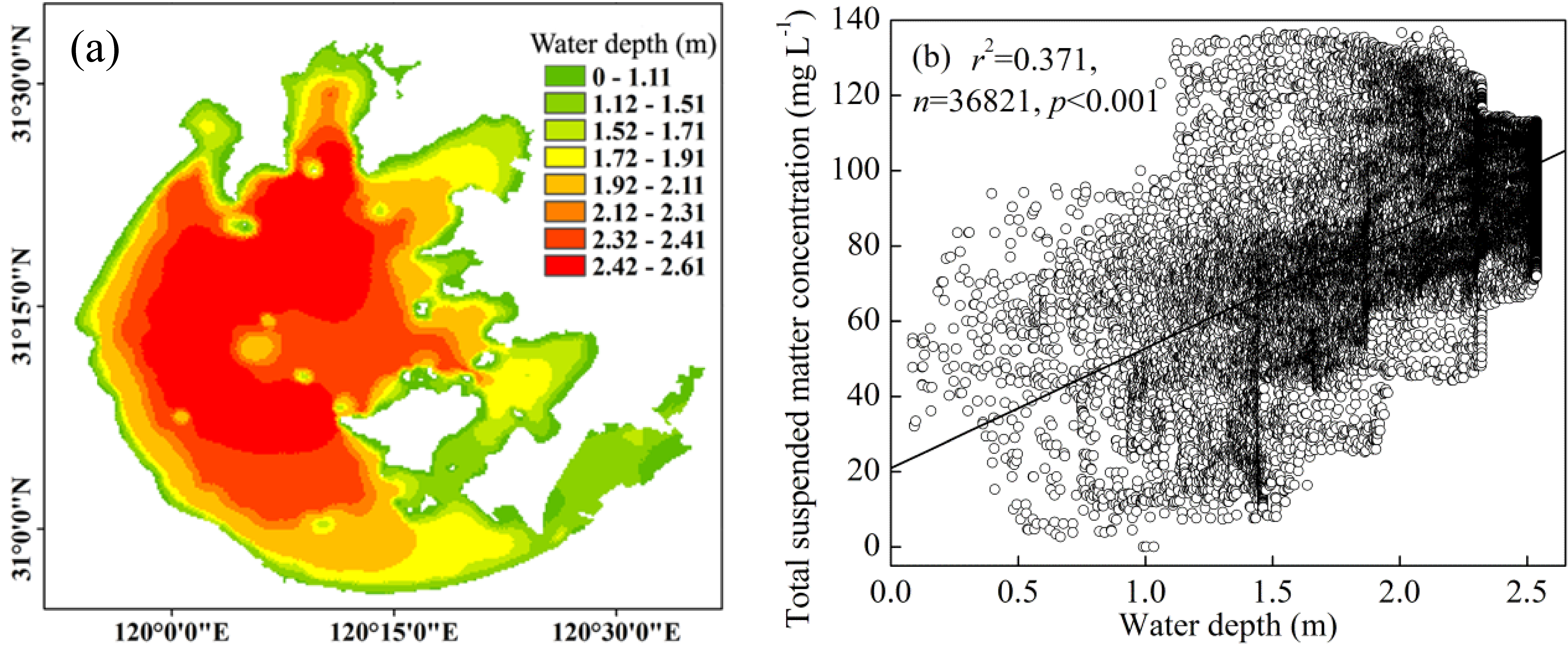

Supplement: Figure S3 — Spatial distribution of water depth in Lake Taihu (a), and linear relationship between water depth and TSM (b). (TIF) [file pone.0098055.s003.tif]

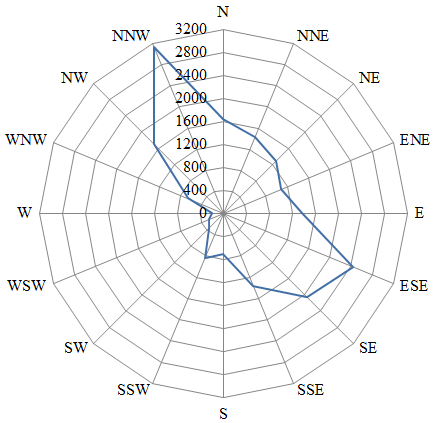

Supplement: Figure S4 — Rose diagram frequency distribution of 16 wind directions of daily maximal wind speed from January 1956 to August 2013 at Dongshan meteorogical station. (TIF) [file pone.0098055.s004.tif]
