# Supplementary material for: Lake Topography and Wind Waves Determining Seasonal-Spatial Dynamics of Total Suspended Matter in Turbid Lake Taihu, China: Assessment Using Long-Term High-Resolution MERIS Data
Source: PLoS One. 2014 May 20;9(5):e98055. doi: 10.1371/journal.pone.0098055 (PMC4028274; doi:10.1371/journal.pone.0098055)
Supplement: Table S1 — Cruise sampling date, number of samples, distribution of sampling sites, type of data, and MERIS data date for 14 cruises in Lake Taihu during 2005–2010. (DOCX) [file pone.0098055.s005.docx]

Table S1. Cruise sampling date, number of samples, distribution of sampling sites, type of data, and MERIS data date for 14 cruises in Lake Taihu during 2005-2010.

| Cruise sampling date | Number of samples | Distribution of sampling sites | Type of data | MERIS data date |
| --- | --- | --- | --- | --- |
| 2005  17 January | 14 | Meiliang Bay, Zhushan Bay, Gonghu Bay, and lake center | w | 17 January |
| 20-22 February | 26 | Entire lake | w | 21 February |
| 17-19 November | 21 | Entire lake | w | 17 November |
| 17 December | 12 | Meiliang Bay, Zhushan Bay, Gonghu Bay, and lake center | w | 17 December |
| 2006  7-9 January | 47 | Entire lake | w, rs | / |
| 29 July-1 August | 47 | Entire lake | w, rs | / |
| 12-15 October | 43 | Entire lake | w, rs | / |
| 2007  7-9 January | 49 | Entire lake | w, rs | 9 January |
| 25-27 April | 45 | Entire lake | w, rs | / |
| 8-21 November | 53 | Entire lake | w, rs | 20, 21 November |
| 19-21 November | 23 | Entire lake | w | 20 November |
| 2008  10-21 November | 63 | Entire lake | w, rs | 20 November |
| 2009  13 January | 8 | Meiliang Bay, Zhushan Bay, Gonghu Bay, and lake center | w | 13 January |
| 2010  14 January | 12 | Meiliang Bay, Zhushan Bay, Gonghu Bay, and lake center | w | 14 January |

w, water sample collection; rs, remote sensing reflectance measurement. There were a total of 347 sites from which water samples were collected and in situ remote sensing reflectance was measured. There are a total of 147 sites at which there were synchronous water sample collections and MERIS images.
