# Supplementary material for: Lake Topography and Wind Waves Determining Seasonal-Spatial Dynamics of Total Suspended Matter in Turbid Lake Taihu, China: Assessment Using Long-Term High-Resolution MERIS Data
Source: PLoS One. 2014 May 20;9(5):e98055. doi: 10.1371/journal.pone.0098055 (PMC4028274; doi:10.1371/journal.pone.0098055)
Supplement: Table S2 — Seasonal distribution of MERIS images in Lake Taihu during 2003–2011. The dates of the images are shown in 8 digits: year, month, and date. (DOCX) [file pone.0098055.s006.docx]

Table S2. Seasonal distribution of MERIS images in Lake Taihu during 2003-2011. The dates of the images are shown in 8 digits: year, month, and date.

| Spring  (*n*=10) | Summer  (*n*=12) | Autumn  (*n*=11) | Winter  (*n*=17) |
| --- | --- | --- | --- |
| 20050331, 20050512,  20060316, 20060417, 20080424, 20080425, 20080501, 20090409, 20090425, 20100429 | 20030805, 20030824, 20040704, 20040814, 20040830, 20050727, 20050809, 20050815, 20060731, 20060803  20070814, 20110724 | 20031023, 20040918, 20041102, 20041105,  20050919, 20051008,  20071111, 20071120,  20081120, 20091124, 20111121 | 20040205, 20050117, 20050120, 20051217, 20061202, 20061218, 20070109, 20070211, 20080214, 20081206, 20081209, 20081225, 20090110, 20090113, 20100114, 20110224, 20111210 |
